# Biomechanical Impact of the Sclera on Corneal Deformation Response to an Air-Puff: A Finite-Element Study

**DOI:** 10.3389/fbioe.2018.00210

**Published:** 2019-01-10

**Authors:** B. Audrey Nguyen, Cynthia J. Roberts, Matthew A. Reilly

**Affiliations:** ^1^Department of Biomedical Engineering, The Ohio State University, Columbus, OH, United States; ^2^Department of Ophthalmology & Visual Science, The Ohio State University, Columbus, OH, United States

**Keywords:** sclera, biomechanics, air-puff, finite-element, deformation

## Abstract

**Aim or Purpose:** To describe the effect of varying scleral stiffness on the biomechanical deformation response of the cornea under air-puff loading via a finite-element (FE) model.

**Methods:** A two-dimensional axisymmetric stationary FE model of the whole human eye was used to examine the effects varying scleral stiffness and intraocular pressure (IOP) on the maximum apical displacement of the cornea. The model was comprised of the cornea, sclera, vitreous, and surrounding air region. The velocity and pressure profiles of an air-puff from a dynamic Scheimpflug analyzer were replicated in the FE model, and the resultant profile was applied to deform the cornea in a multiphysics study (where the air-puff was first simulated before being applied to the corneal surface). IOP was simulated as a uniform pressure on the globe interior. The simulation results were compared to data from *ex vivo* scleral stiffening experiments with human donor globes.

**Results:** The FE model predicted decreased maximum apical displacement with increased IOP and increased ratio of scleral-to-corneal Young's moduli. These predictions were in good agreement (within one standard deviation) with findings from *ex vivo* scleral stiffening experiments using human donor eyes. These findings demonstrate the importance of scleral material properties on the biomechanical deformation response of the cornea in air-puff induced deformation.

**Conclusion:** The results of an air-puff induced deformation are often considered to be solely due to IOP and corneal properties. The current study showed that the stiffer the sclera, the greater will be the limitation on corneal deformation, separately from IOP. This may have important clinical implications to interpreting the response of the cornea under air-puff loading in pathologic conditions.

## Introduction

Biomechanical markers are being explored to improve screening, diagnosis, and management of diseases such as keratoconus and glaucoma (Liu and Roberts, 2005; Elsheikh et al., 2009; Ruberti et al., 2011; Coudrillier et al., 2012; Tang and Liu, 2012; Hon and Lam, 2013; Metzler et al., 2014; Girard et al., 2015; Sinha Roy et al., 2015; Ariza-Gracia et al., 2016; Roberts, 2016). Biomechanical properties of the sclera are often determined via *ex vivo* mechanical strip testing or inflation tests (Coudrillier et al., 2012; Geraghty et al., 2012; Girard et al., 2015; Pandolfi and Boschetti, 2015; Nguyen, 2016). However, this destructive method depends on a multitude of factors including tissue hydration and strain rate, and there is a wide range of reported values in the literature on the value of Young's modulus of the sclera (Coudrillier et al., 2012). There is a demonstrated need for a clinical tool to determine the *in vivo* biomechanical behavior of ocular tissues, including how the cornea and the sclera interact.

It has been shown that the biomechanical response of the cornea under air-puff deformation is significantly affected by its boundary properties (Elsheikh, 2010; Metzler et al., 2014). Metzler et al. showed that the human cornea behaves more stiffly in the case of a corneoscleral button mounted on a rigid artificial anterior chamber (simulating stiffer scleral material properties) as opposed to a cornea that remains connected to an intact whole globe at physiologically normal values of IOP. Their results suggest that the scleral properties have an important impact on the deformation response of the cornea under air-puff loading (Metzler et al., 2014).

Two commercial devices have been developed to assess the biomechanical deformation response of the cornea using an air-puff as the non-destructive load, and they have been used to provide clinical insight into the *in vivo* biomechanical behavior in pathological conditions (Luce, 2005; Ambrósio et al., 2013). The data produced by these devices are often interpreted as purely corneal response without considering the contribution of the sclera. Therefore, our purpose is to develop a whole-eye finite-element model that utilizes a well-characterized air-puff as the load to investigate the impact of varying scleral properties on corneal deformation response. This model will be validated using *ex vivo* experimental data obtained using human donor eyes.

## Methods

The CorVis ST (Oculus Optikgeräte, GmbH, Wetzlar, Germany) utilizes a high-speed camera with Scheimpflug geometry at a frame rate of 4,300 Hz to acquire 140 images of the cornea along a single horizontal meridian, providing dynamic corneal deformation response parameters, and visualization of the cornea as it deforms (Roberts, 2014). Additionally, the air-puff of the CorVis ST has been shown to be consistent and repeatable (Roberts et al., 2017), and for these reasons was chosen as the load for the FE model. The ability to apply an accurate and precise load while monitoring deformation is the hallmark of mechanical testing.

### Geometry and Mesh

**Figure 1:**
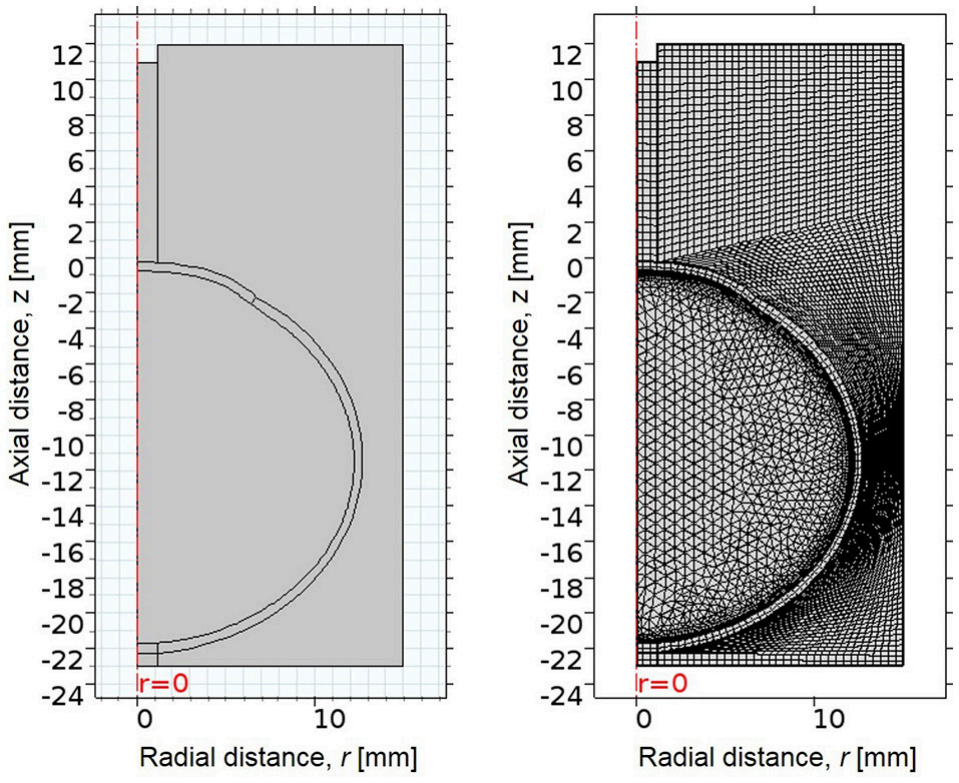
**(Left)** 2D axisymmetric geometry of eye, consisting of cornea, sclera, and vitreous, surrounded by an air region. The x-axis indicates radial distance from the line of rotational symmetry, and the y-axis indicates axial distance, where the undeformed corneal apex is at 0 mm. **(Right)** The mesh of the FE model, consisting of quadrilateral and free triangular elements.

A simplified axisymmetric FE model of the human eye was developed in COMSOL Multiphysics 5.2a (COMSOL Inc.; Burlington, MA) based on average dimensions (Figure 1, Table 1). The whole-eye geometry consisted of cornea, sclera, and vitreous humor. The whole globe has a diameter of 24 mm, the cornea has a diameter of 11 mm, and the central corneal thickness (CCT) is 500 μm. The thickness of the sclera ranged from 400 to 1,000 μm.

**Table 1:** Summary of geometric parameters for finite element model.

**Tissue**	**Geometric parameter**	**Parameter value**
Cornea	Central corneal thickness	500 μm (Elsheikh and Wang, 2007)
	Anterior radius of curvature	8.0 mm (Vojniković et al., 2013)
	Posterior radius of curvature	6.8 mm (Vojniković et al., 2013)
Sclera	Thickness at equator	400 μm (Norman et al., 2010)
	Thickness at posterior pole	1,000 μm (Norman et al., 2010)
	Radius of curvature	12 mm (Norman et al., 2010)

**Figure 2:**
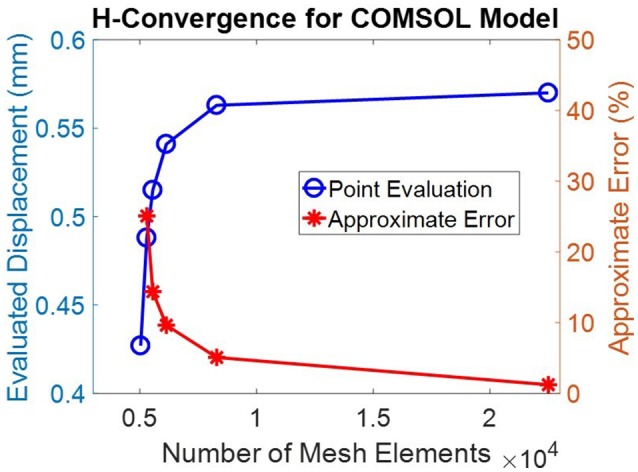
A convergence study showed that a mesh containing >20,000 elements was sufficient to achieve discretization errors which were significantly less than normal experimental variations. This mesh density, shown in Figure 1, was therefore adopted for all subsequent simulations.

The geometry was meshed using the built-in meshing capabilities of COMSOL. The central air region and ocular tunic were meshed with mapped 12,837 quadrilateral elements, with 3 elements through the thickness of the cornea and sclera. The remaining air region domains were meshed with 7,453 free triangular elements. The globe interior was meshed using free triangular elements and boundary layers to create a highly refined mesh at the interface of the ocular tunic and the vitreous body and more accurately model corneal deformation response. A convergence study was undertaken to identify the mesh density required to ensure that the solution was nearly independent of meshing parameters (Figure 2).

### Estimation of Stress-Free Geometry

**Figure 3:**
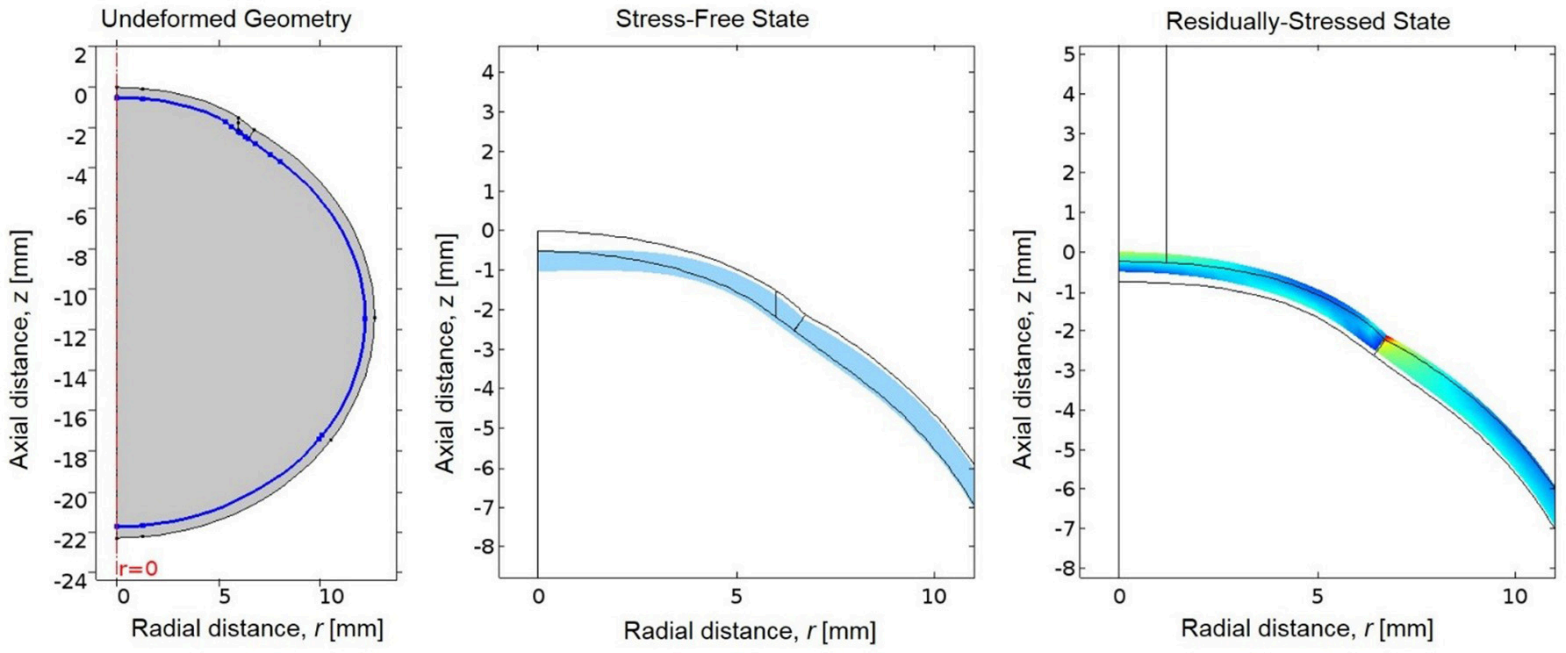
**(Left)** The undeformed geometry of the eye, where the interior boundary of the ocular tunic is highlighted in blue to indicate where the negative IOP will be applied to estimate the stress-free geometry of the eye. **(Center)** The resultant stress-free state of the eye (in blue) compared to the original undeformed geometry prior to loading by the negative IOP (in black). **(Right)** Von Mises stresses in the residually-stressed state of the eye when loaded by IOP, where the black lines represent the geometry of the stress-free state of the eye prior to loading by IOP.

The eye is naturally under tension due to loading by intraocular pressure (IOP); therefore the unloaded or stress-free state of the eye was first estimated (Elsheikh et al., 2013). A negative pressure equal to the IOP was applied to the interior boundaries of the ocular tunic to generate the unloaded state. The resultant stress-free geometry was then loaded by IOP to determine the residually-stressed state of the eye (Figure 3). This residually-stressed geometry was used as the starting point for the multiphysics FE simulation.

### Material Properties and Boundary Conditions

The multiphysics model included the air-puff from the CorVis ST, which acted as the anterior load on the eye. The air-puff velocity and pressure profiles acting on the anterior cornea were simulated using turbulent k-ω shear stress transport fluid flow physics. The maximum velocity at the nozzle outlet was set to 140.2 m/s based on data from the characterization performed by Roberts et al. (2017). The remaining edges of the air region were set to be open boundaries, such that the air flow is not affected by the arbitrary region boundaries. The resultant pressure profile from the air-puff simulation was applied as the load on the anterior cornea to simulate corneal deformation. The central 1.5 mm radius of the posterior sclera was held fixed.

The material properties of the ocular tunic were described by an isotropic, nearly incompressible, neo-Hookean constitutive model with values estimated from the literature (Heys et al., 2001; Ng and Ooi, 2006; McKee et al., 2011). The vitreous was modeled as a linear elastic solid (Heys et al., 2001). Table 2 summarizes the material properties utilized in the FE model. The air-puff region surrounded the eye, which was fixed at the back to replicate the mounted whole-globe setup used in the *ex vivo* validation studies. The Young's modulus of the cornea was fixed at a representative value from the literature (Hamilton and Pye, 2008; McKee et al., 2011) while the Young's modulus of the sclera was varied to examine its relative effect on corneal deformation (Heys et al., 2001; Ng and Ooi, 2006). Intraocular pressure (IOP) was varied from 10 to 40 mmHg in 10 mmHg increments. The properties of air, cornea, and vitreous were kept constant between simulations.

**Table 2:** Material properties of ocular tissues in the FE model.

	**Cornea**	**Sclera**	**Vitreous**	**Air**
Young's modulus [MPa]	1.5 (Hamilton and Pye, 2008; McKee et al., 2011)	2.25, 3.0, 4.5, 6.0 (McKee et al., 2011)	–	–
Poisson ratio	0.49	0.49	0.49	–
Bulk modulus [MPa]	50.68	76.01	0.375 (Heys et al., 2001)	–
Shear modulus [Pa]	–	–	0.75 (Heys et al., 2001)	
Density [kg/m^3^]	1,050 (Ng and Ooi, 2006)	1,100 (Ng and Ooi, 2006)	1,000 (Heys et al., 2001)	1.1855
Viscosity [Pa^*^s]	–	–		18.6E-6

In the *ex vivo* setup of human donor studies, which was replicated by the finite element model, the peak of corneal motion/deformation is in phase with the peak of the air-puff loading curve. As the loading rate (dP/dt) approaches 0, this aligns with the maximum apical displacement as measured by the CorVis ST. We therefore assumed, as a first approximation, the cornea is in a quasi-static equilibrium such that a static model will capture this particular aspect of the air-puff-induced deformation.

### *Ex vivo* Experiments

The CorVis ST was used to acquire data on the biomechanical deformation response of the corneas of 12 pairs of human donor eyes (65 ± 11.4 years, 8 male/4 female). The donor eye study is considered exempt from review by the university. In each pair, one eye was randomly selected to have its sclera stiffened by crosslinking treatment with glutaraldehyde, while the fellow eye served as a control. The globe was immersed for 30 min in 4% glutaraldehyde in Dulbecco's phosphate-buffered saline (DPBS) (Sigma-Aldrich, St. Louis, Missouri, USA) just below the level of the limbus, leaving a visible gap such that the cornea remained untreated. Glutaraldehyde visibly stains ocular tissues yellow after a short period of time. The ocular tissues were visually inspected to ensure that the cornea remained unaffected by the glutaraldehyde treatment.

**Figure 4:**
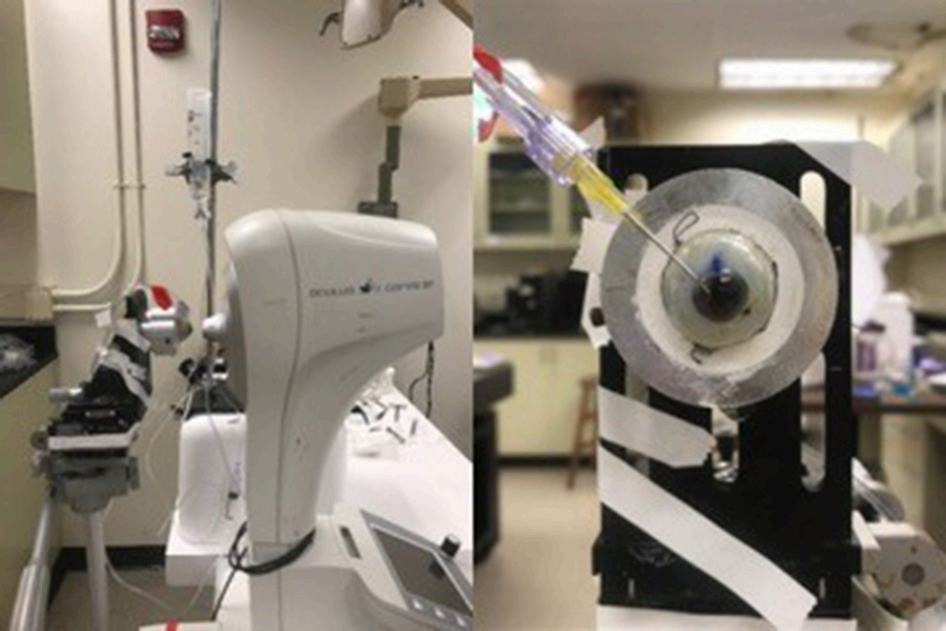
**(Left)** Experimental setup for *ex vivo* studies on human donor eyes, showing a whole globe in the purpose-designed mount in front of the CorVis ST. **(Right)** A view of the human donor eye showing the 22-gauage needle inserted into the anterior chamber, used to set and maintain intraocular pressure.

The CorVis ST was used to load the eye and quantify the resulting corneal deformation response (Figure 4). Each eye was secured in a custom whole-globe mount using shallow sutures in the sclera. A 22-gauge needle attached to a saline column was inserted into the anterior chamber of the eye to set and maintain IOP. The column was set to specified heights to generate IOPs corresponding to 10, 20, 30, and 40 mmHg. At least 3 examinations were performed at each pressure step, with DPBS dripped onto the cornea between examinations to maintain hydration. All data were acquired within 48 h post-mortem.

## Results

**Figure 5:**
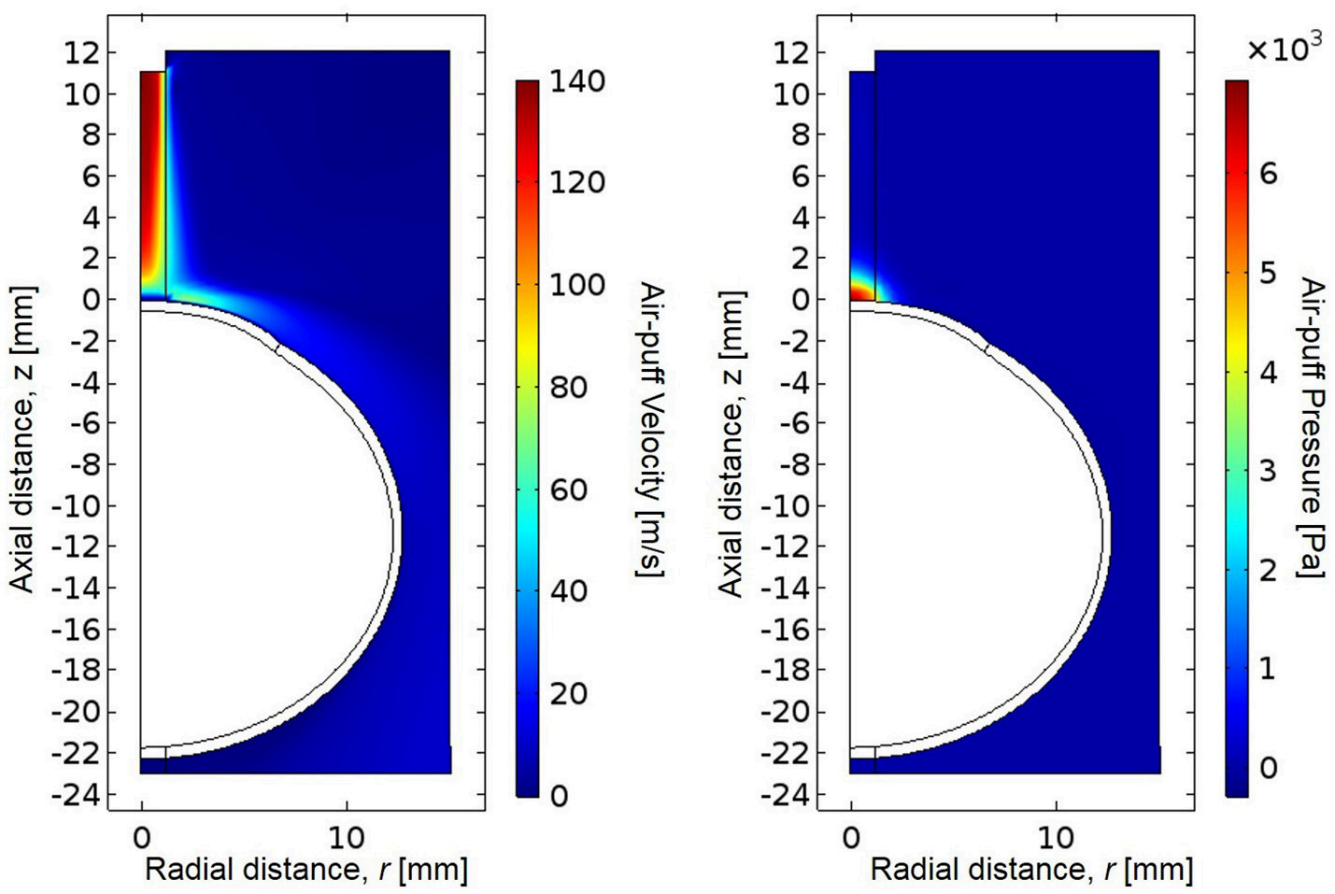
Simulated CorVis ST air-puff velocity profile **(left)** and pressure profile **(right)**, which serves as the load on the anterior boundary of the globe. The simulation profiles agree within 2% of experimental measurements using hot-wire anemometry from Roberts et al. (2017).

The CorVis ST air-puff velocity profile and corresponding pressure profile were simulated in COMSOL 5.2a (Figure 5). The spatial velocity profile agreed within 2% of experimental measurements by hot wire anemometry (Roberts et al., 2017) along the centerline/axis of symmetry as well as the central corneal region (Tables 3, 4). The coordinate system for the model has the undeformed corneal apex at the origin, where r indicates the radial distance from the axis of symmetry, and z indicates the axial distance from the undeformed corneal apex. The air-puff pressure is concentrated at the central corneal region.

**Table 3:** Spatial measurements of air flow velocity at 4 mm from nozzle at peak pressure.

**Radial distance, r [mm]**	**Simulation [m/s]**	**Hot wire (Roberts et al., 2017) [m/s]**	**Error [%]**
0.75	120.496	120.734	−0.196
1.50	50.601	50.594	0.015

**Table 4:** Spatial measurements of air flow velocity along axis of symmetry at peak pressure.

**Axial distance, z [mm]**	**Simulation [m/s]**	**Hot wire (Roberts et al., 2017) [m/s]**	**Error [%]**
11	140.524	140.218	0.218
9	138.394	137.725	0.486
7	136.440	137.609	−0.850
5	135.186	136.697	−1.105
3	134.616	135.337	−0.533
1	134.178	133.238	0.706

**Figure 6:**
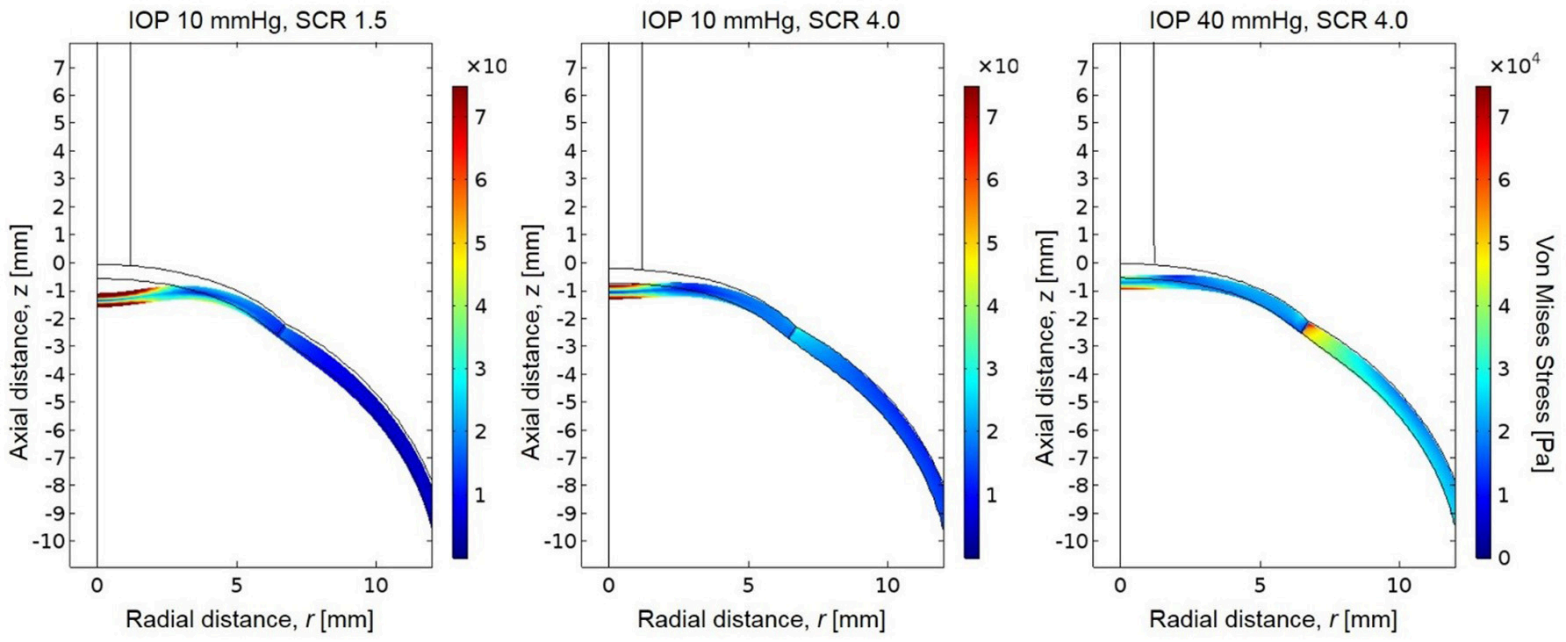
Resultant von Mises stresses and deformation for a globe with scleral-to-corneal ratio of Young's moduli of 1.5 and IOP of 10 mmHg **(left)**, scleral-to-corneal ratio of 1.5 and IOP of 40 mmHg **(center)**, and a globe with scleral-to-corneal ratio of 4.0 and IOP of 40 mmHg **(right)**.

The simulated pressure profile was applied as the boundary load on the anterior boundary of the eye. The resulting stress magnitudes were highest at the posterior surface of the corneal apex, which experiences the greatest total deformation and strain (Figure 6). The stresses are also higher at the posterior sclera where the eye was fixed in this simulation. There is an increase in the magnitude of stress near the area of the limbus (interface of cornea and sclera) due to the dissimilar stiffnesses between cornea and sclera in the model. A sensitivity analysis was performed to show model independence from the vitreous body properties (Table 5).

**Table 5:** Sensitivity analysis of model dependence and vitreous body.

**Vitreous shear modulus [Pa]**	**Apical displacement [mm]**	**Percent change from 7.5 Pa [%]**
0.75000	−0.81888	0.147
7.5000	−0.81768	–
75.000	−0.81002	0.937

**Figure 7:**
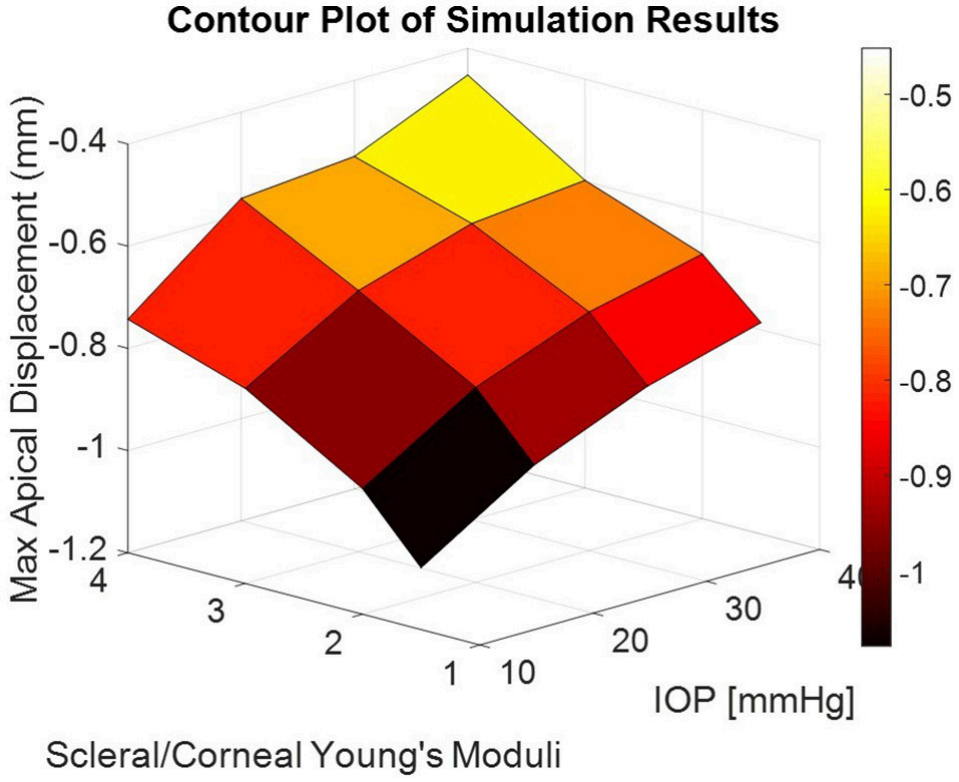
Contour plot of maximum apical displacement predicted by the finite-element model as a function of IOP and scleral-to-corneal ratio of Young's moduli. The biomechanical response is non-linear with both increasing IOP and increasing sclera-to-corneal ratio.

**Figure 8:**
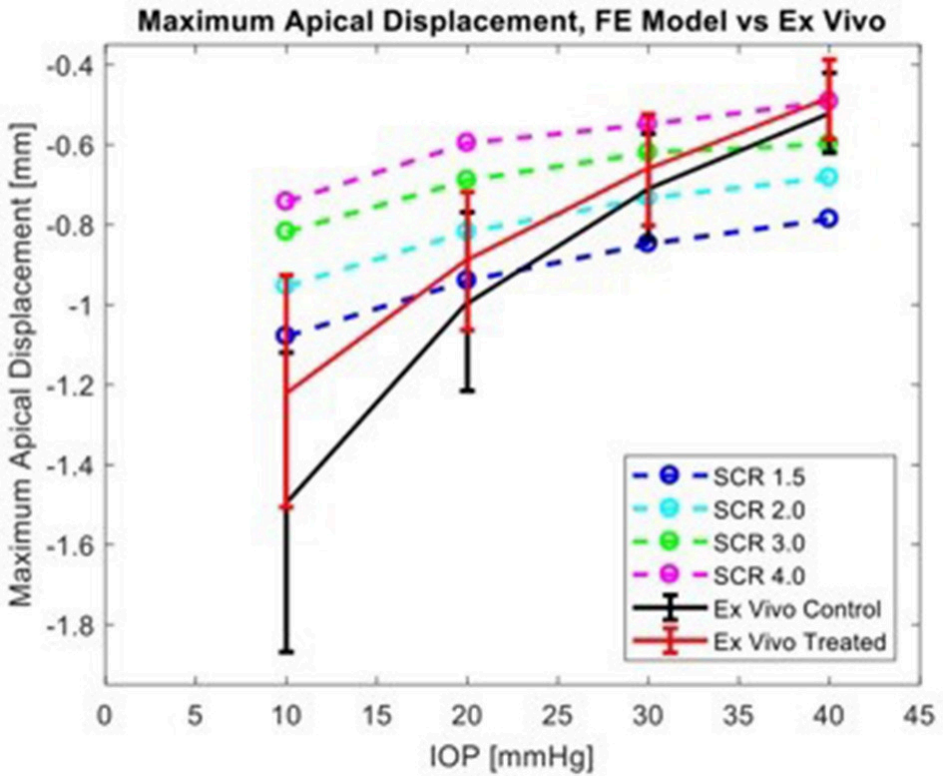
Comparison of simulation results from FE model for several levels of scleral-to-corneal ratio of Youngs' Modulus (SCRs) against *ex vivo* data. Error bars represent 95% confidence intervals.

The corneal deformation response produced by the model matched the expected trend (Table 6). For each value of IOP tested, increasing the ratio of scleral to corneal Young's moduli resulted in decreasing maximum apical displacement (Figure 7). Further, increasing the value of IOP while keeping other material properties constant also resulted in decreasing maximum apical displacement, which is consistent with with the results of our *ex vivo* experiments (Table 7). Figure 8 shows that the effective stiffness of the cornea increases with higher IOP, resulting in decreased maximum apical displacement in both the FE model and *ex vivo* results. The simplified Nehookean approximation may contribute to the why the model does not completely bound *ex vivo* observations.

**Table 6:** Summary of simulation results from FE model.

**IOP [mmHg]**	**Scleral-to-corneal ratio [MPa/MPa]**	**Max apical displacement [mm]**
10	1.5	−1.079
	2	−0.954
	3	−0.818
	4	−0.743
20	1.5	−0.940
	2	−0.818
	3	−0.689
	4	−0.569
30	1.5	−0.848
	2	−0.733
	3	−0.620
	4	−0.549
40	1.5	−0.786
	2	−0.682
	3	−0.598
	4	−0.492

**Table 7:** Summary of *ex vivo* results from human donor eyes (Nguyen et al., 2018).

**IOP [mmHg]**	**Group**	**Max apical displacement [mm]**
		**Mean ± Std. Dev**.
10	Control	−1.494 ± 0.191
	Treated	−1.216 ± 0.148
20	Control	−0.992 ± 0.114
	Treated	−0.891 ± 0.088
30	Control	−0.706 ± 0.068
	Treated	−0.663 ± 0.071
40	Control	−0.520 ± 0.051
	Treated	−0.487 ± 0.051

## Discussion

Scleral stiffness significantly influenced corneal apical displacement in both simulations and *ex vivo* experiments. Thus, with increasingly stiff sclera, the greater the limitation on corneal deformation to an air-puff at physiologic levels of IOP. The *ex vivo* results are consistent with the trends observed by Metzler et al., where the untreated globe undergoes stress stiffening at higher IOP and behaves similarly to the treated globe. This has important clinical implications, where the corneal biomechanical response is often attributed solely to the corneal properties. The maximum apical displacement of the cornea decreased non-linearly with increasing IOP, which is consistent with the principles of tonometry and literature reports (Metzler et al., 2014). Because traditional methods for biomechanical evaluation are limited to *ex vivo* studies, finite-element (FE) models have been explored as a way to evaluate *in vivo* properties and response (Elsheikh, 2010; Girard et al., 2015; Pandolfi and Boschetti, 2015; Ariza-Gracia et al., 2016). In published models that simulate corneal deformation by an air-puff, some do not include the sclera (Elsheikh et al., 2009, 2013; Kling et al., 2014; Lago et al., 2014; Pandolfi and Boschetti, 2015; Sinha Roy et al., 2015; Bekesi et al., 2016; Simonini and Pandolfi, 2016). The inseparable impact of the sclera to limiting corneal deformation response requires a whole-eye model. Further, the simulated load must be accurate because the biomechanical response is load-dependent.

This FE model, while simple, achieved qualitative agreement with *ex vivo* experiments. Several aspects of this result give insights which can be used to inform other models. Several models of air puff-induced deformation are comprised of only the cornea, with a variety of scleral boundary conditions applied at the limbus (Elsheikh et al., 2009, 2013; Kling et al., 2014; Lago et al., 2014; Pandolfi and Boschetti, 2015; Sinha Roy et al., 2015; Bekesi et al., 2016; Simonini and Pandolfi, 2016). Our findings indicate that this simplifying representation of the scleral influence on the limbus is inaccurate and introduces systematic errors in these models. Non-contact tonometry devices estimate IOP by observing the response of the cornea; our findings demonstrate that clinical interpretations which neglect scleral contributions to the corneal response to an air-puff may lead to incorrect conclusions. This is particularly important in conditions where the sclera is altered, such as prostaglandin treatment in glaucoma (Toris et al., 2008; Alm and Nilsson, 2009)—which increases scleral permeability and uveoscleral outflow—and progressive myopia (McBrien and Gentle, 2003; Harper and Summers, 2015)—in which the sclera experiences significant collagen remodeling. Reductions in IOP have been reported in some procedures that alter the sclera to treat presbyopia; this is likely artifact from the altered sclera, and there is little or no actual change in IOP (Fukasaku and Marron, 2001; Hipsley et al., 2017).

The present model accurately replicated the velocity and pressure profiles of the CorVis ST air-puff. Similar to the work done by Elsheikh et al. the model also estimated the stress-free state of the eye and generated the residually-stressed state before deformation to more accurately describe the stresses in the cornea and sclera and the resultant biomechanical response (Elsheikh et al., 2013). Finally, a significant advantage of this model is that the simulation results are qualitatively validated with data from *ex vivo* human donor eyes, in an experimental setup that was replicated closely in the FE model.

One limitation of this model is that it does not account for the dynamic time-dependent response of the cornea to the air-puff. The cornea is a soft biological tissue and therefore exhibits viscoelasticity in its biomechanical response. To account for the non-linear behavior of the tissue, we chose to consider the ocular tissues as hyperelastic, resulting in a non-linear response to increasing IOP. The use of an incompressible neo-Hookean constitutive model is a simplification: the cornea and sclera are complex structures with significant anisotropy (Nguyen, 2016). In this case, the model effectively assumed that the secant modulus is constant regardless of the magnitude of deformation. It is therefore not surprising that the experimental response shows a much larger dependence on SCR and behaves in a strain-stiffening manner. Inclusion of more accurate material models, such as Mooney-Rivlin, will significantly improve the model's ability to make quantitative predictions. Another limitation of this model it does not capture the dynamic nature of the air-puff test. Despite this simplification, the steady-state approximation produced results which qualitatively matched those from *ex vivo* experiments on human donor tissues. Future iterations of the model will include the dynamic response of the cornea to an air-puff.

The finite-element model presented in this paper demonstrates that scleral material properties have an important impact on the biomechanical deformation response of the cornea in air-puff induced deformation. Namely, the stiffer the sclera, the greater the limitation on corneal deformation. This may have important clinical implications. Often in the clinic, the observed biomechanical deformation response of the cornea is attributed solely to the material properties of the cornea, as well as IOP. However, it is clear from these simulations and experiments that the deformation response of the same cornea varies significantly with varying scleral properties. This suggests that when looking at air-puff induced deformation, that the observed biomechanical response is a result of the combination of both corneal and scleral material properties, in addition to IOP.

## Data Availability Statement

The datasets analyzed for the current study are available from the corresponding author on reasonable request.

## Author Contributions

BN, MR, and CR contributed to this study. BN and CR conceived the study design and all authors contributed to the development of the model. All authors contributed to manuscript revision, read and approved the submitted version.

### Conflict of Interest Statement

CR is a consultant for Oculus Optikgeräte GmbH. The remaining authors declare that the research was conducted in the absence of any commercial or financial relationships that could be construed as a potential conflict of interest.
